# Telangiectatic Matting as a Complication After Aesthetic Procedures: A Case Report

**DOI:** 10.7759/cureus.108372

**Published:** 2026-05-06

**Authors:** Giovanna Cabral, Nayara Batagini, Antonio Zerati, Andre Estenssoro, Yuri Oliveira, Brenno Augusto S Mello Netto

**Affiliations:** 1 Vascular Surgery, Clinica Giovana Cabral, São Paulo, BRA; 2 Vascular Surgery, Hospital Sirio Libanes, São Paulo, BRA; 3 Vascular Surgery, Beneficência Portuguesa (BP) Hospital, São Paulo, BRA; 4 Vascular Surgery, Seabra Excelencia Vascular, Vitória, BRA

**Keywords:** case report, matting, phlebology, s: varicose veins, telangiectasia

## Abstract

Telangiectatic matting is commonly described as a transient complication following sclerotherapy and other vascular interventions. Its occurrence after permanent body fillers has not been clearly established. We report the case of a 27-year-old woman who developed persistent telangiectatic matting in the gluteal region several months after polymethylmethacrylate (PMMA) injection for cosmetic augmentation, without prior sclerotherapy in the affected area. A multimodal therapeutic approach was employed, including transdermal Nd:YAG laser and sclerotherapy, resulting in partial clinical improvement. This case suggests a possible association between permanent fillers and delayed microvascular alterations, highlighting the importance of long-term surveillance after aesthetic procedures.

## Introduction

Aesthetic procedures may be associated with vascular and cutaneous complications, including telangiectasias and microvascular alterations [1]. Among these, telangiectatic matting is most commonly described following vascular treatments, particularly sclerotherapy [2,3]. In most cases, it is self-limited, with spontaneous resolution within six months [4].

Telangiectatic matting may also be related to local inflammatory reactions and hormonal factors, especially estrogen-based therapies [5,6]. Although it has low clinical morbidity, it carries significant aesthetic and psychosocial impact and may represent a therapeutic challenge due to its often limited response to available treatment modalities [7].

Concurrently, there has been a global increase in the performance of aesthetic procedures, both surgical and minimally invasive. Brazil occupies a leading position worldwide in this field, with growing demand for facial and body fillers [8,9]. Among these materials, polymethylmethacrylate (PMMA) stands out due to its permanent nature, providing long-lasting results but also associated with a higher potential for late adverse events [8,9].

PMMA is a synthetic, non-absorbable material capable of inducing chronic inflammatory responses and foreign-body reactions, including granuloma formation, tissue necrosis, infections, vascular embolization, and persistent microvascular alterations [8-10]. Although these complications are documented, late microvascular repercussions remain poorly explored in the literature. To date, there are no consistent reports describing persistent telangiectatic matting following body filler procedures with PMMA outside the classical context of vascular treatments. Therefore, the present study aims to report an unprecedented case of this complication.

## Case presentation

A 27-year-old female patient, Fitzpatrick skin type II, non-smoker, physically active, with no comorbidities, no drug allergies, and no history of hormonal contraceptive use, presented to a private vascular clinic with an aesthetic complaint. She exhibited combined telangiectasias in the gluteal region, more pronounced on the left side, which developed after a body contouring procedure involving the injection of approximately 160 mL of polymethylmethacrylate (PMMA) three years prior.

**Figure 1:**
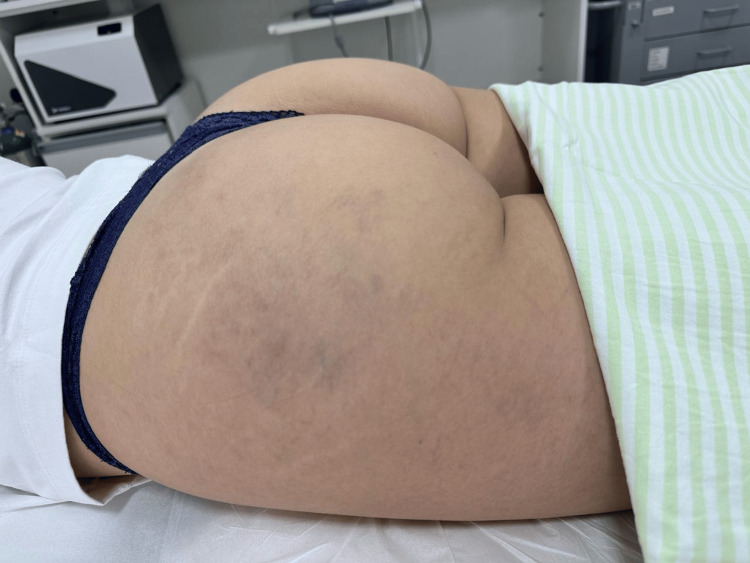
Pre-treatment clinical image demonstrating diffuse telangiectatic matting in the lateral gluteal region.

At the initial evaluation, diffuse telangiectatic matting was observed in the treated area. According to the patient, the lesions had appeared more than six months after the filler procedure and persisted without spontaneous resolution. The initial clinical presentation is shown in Figure 1.

The patient was instructed to maintain adequate skin hydration and was prescribed diosmin plus hesperidin (1000 mg/day) and pycnogenol (100 mg/day) orally throughout the treatment period. Clinical photographs were obtained. A treatment plan consisting of three sessions of combined therapy and a final follow-up visit was proposed.

The patient signed an informed consent form detailing the proposed treatments and potential complications.

During the first session, transdermal Nd:YAG 1064 nm laser was performed using parameters 6/15/80 and 3/15/175, combined with 75% hypertonic glucose sclerotherapy. After 45 days, minimal improvement was observed. At that time, foam sclerotherapy with 0.25% polidocanol was performed, associated with tumescent anesthesia (START technique, as described by Ramelet [4]) and transdermal Nd:YAG 1064 nm laser with parameters 3/15/150. A satisfactory visual response was noted. Microneedling was also performed in the same session for more erythematous lesions.

**Figure 2:**
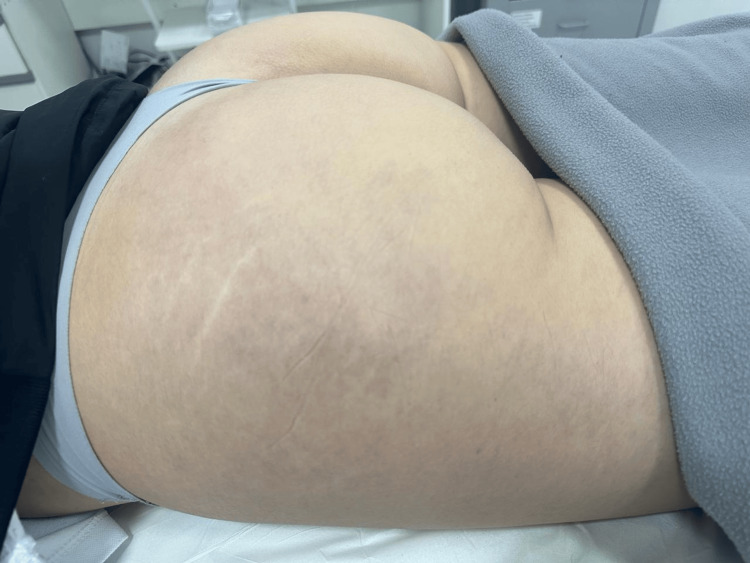
Clinical appearance after the third treatment session, demonstrating partial reduction in telangiectatic density and decreased erythematous background in the lateral gluteal region.

The third visit was scheduled for 60 days later, but occurred four months after the second session due to patient-related factors. Nd:YAG 1064 nm laser monotherapy was performed using parameters 6/20/70 and 3/15/175. Post-treatment findings after the third session are demonstrated in Figure 2.

The patient was subsequently lost to follow-up. Telephone contact revealed that she had relocated to another city and considered the treatment satisfactory, reporting clinical improvement.

## Discussion

Telangiectatic matting is characterized by the development of a network of fine, superficial telangiectasias, usually reddish or violaceous, occurring most frequently after sclerotherapy procedures [2-4]. It is a well-recognized complication in phlebology, particularly in young women, although its pathophysiology is not fully understood. Proposed mechanisms include persistent vasodilation, reactive angiogenesis, local inflammation, and alterations in cutaneous vasomotor control [3,4].

The main predisposing factors described in the literature include female sex, thin skin, prior telangiectasias, hormonal influences, genetic predisposition, and local vascular trauma [2,3,5,6]. In most cases, telangiectatic matting is self-limited; however, in certain situations, it may persist and demonstrate limited response to treatment, representing both a clinical and aesthetic challenge [1,4].

PMMA functions as a permanent implant, remaining indefinitely in the tissues and inducing a chronic low-grade inflammatory response [8,9]. This process may lead to continuous release of inflammatory mediators, persistent macrophage activation, disorganized neoangiogenesis, and microcirculatory alterations, mechanisms already implicated in other complications related to permanent fillers [9-11].

Furthermore, the presence of non-absorbable particles may interfere with the physiological balance between vasoconstriction and vasodilation, favoring the persistence of telangiectasias and local vasomotor alterations [8,11]. Although complications such as granulomas, tissue necrosis, and infections are more commonly reported after PMMA use, late microvascular alterations, as observed in this case, may represent an underrecognized adverse effect [9-11].

This report differs from previously described cases due to the absence of prior sclerotherapy in the affected area, the delayed onset of matting more than six months after the aesthetic procedure, and the prolonged persistence of lesions without spontaneous resolution [3,4]. These findings suggest that PMMA may act as an independent triggering factor for the development of telangiectatic matting outside the classical vascular treatment context.

This study has limitations inherent to a single case report, including the lack of histopathological confirmation and incomplete long-term follow-up. Nevertheless, its unprecedented nature contributes to expanding current knowledge regarding possible late microvascular complications associated with permanent fillers, particularly PMMA [8-11].

## Conclusions

This report describes an uncommon presentation of persistent telangiectatic matting following an aesthetic procedure with a permanent polymethylmethacrylate-based filler. Although telangiectatic matting is classically associated with vascular treatments, its occurrence in the context of permanent aesthetic fillers broadens the spectrum of potential complications related to these interventions.

The unprecedented nature of this case highlights a gap in current knowledge regarding late microvascular repercussions associated with PMMA use. The significant aesthetic impact and recognized therapeutic difficulty of telangiectatic matting underscore the importance of long-term follow-up, careful patient selection, and the development of more effective and safer therapeutic strategies.
